# Collagen Scaffolds with Controlled Insulin Release and Controlled Pore Structure for Cartilage Tissue Engineering

**DOI:** 10.1155/2014/623805

**Published:** 2014-02-25

**Authors:** Himansu Sekhar Nanda, Shangwu Chen, Qin Zhang, Naoki Kawazoe, Guoping Chen

**Affiliations:** ^1^Tissue Regeneration Materials Unit, International Centre for Materials Nanoarchitectonics, National Institute for Materials Science, 1-1 Namiki, Tsukuba, Ibaraki 305-0044, Japan; ^2^Department of Materials Science and Engineering, Graduate School of Pure and Applied Sciences, University of Tsukuba, 1-1-1 Tennodai, Tsukuba, Ibaraki 305-8571, Japan

## Abstract

Controlled and local release of growth factors and nutrients from porous scaffolds is important for maintenance of cell survival, proliferation, and promotion of tissue regeneration. The purpose of the present research was to design a controlled release porous collagen-microbead hybrid scaffold with controlled pore structure capable of releasing insulin for application to cartilage tissue regeneration. Collagen-microbead hybrid scaffold was prepared by hybridization of insulin loaded PLGA microbeads with collagen using a freeze-drying technique. The pore structure of the hybrid scaffold was controlled by using preprepared ice particulates having a diameter range of 150–250 **μ**m. Hybrid scaffold had a controlled pore structure with pore size equivalent to ice particulates and good interconnection. The microbeads showed an even spatial distribution throughout the pore walls. *In vitro* insulin release profile from the hybrid scaffold exhibited a zero order release kinetics up to a period of 4 weeks without initial burst release. Culture of bovine articular chondrocytes in the hybrid scaffold demonstrated high bioactivity of the released insulin. The hybrid scaffold facilitated cell seeding and spatial cell distribution and promoted cell proliferation.

## 1. Introduction

Hyaline articular cartilage is composed of abundant chondrocytes and limited progenitor cells sparsely embedded in nonvascular extracellular matrix (ECM). Articular cartilage defects are very difficult to heal due to its limited ability of self-repair and regeneration [1–3]. Such defects if untreated may lead to the serious problem of osteoarthritis, a major clinical problem around the world [4, 5]. Current treatment methods for articular cartilage defects include abrasion arthroplasty, subchondral drilling, osteochondral allografting, and periosteal or perichondral tissue grafting [6, 7]. However none of the treatment methods can reproduce the exact characteristics of a hyaline cartilage for optimal healing of the tissue defects. Therefore, cartilage tissue engineering using porous scaffolds, chondrocytes or human mesenchymal stem cells (hMSCs), and bioactive instructive cues has been evolved as an alternative and promising approach to treat cartilage defects [1–3, 6, 8].

Porous scaffolds prepared from biodegradable polymers have been well studied for their ability to regenerate various types of tissues such as skin, cartilage, and bone [9–14]. Collagen as a natural biomaterial and a component of native extracellular matrix (ECM) is extensively investigated for preparation of porous scaffolds for cartilage tissue engineering [10, 13, 15]. However weak mechanical strength of the scaffolds prepared from collagen remains a major hurdle behind the clinical application. Recently, we have developed collagen porous scaffolds with controlled pore structures as an ideal platform for cartilage tissue regeneration because of its high porosity with good pore interconnection, excellent control over the pore structure, and impressive biomechanical properties [15]. Large scaffolds are often needed for major cartilage defects. Formation of necrotic cores due to nutrient depletion is another problem during *in vitro* cell culture over these three-dimensional (3D) scaffolds. It is difficult for nutrients and essential growth factors to diffuse inside the complex porous network of the 3D construct for proper nourishment of the inner cell mass leading to the formation of necrotic cores [16, 17]. Growth factor and therapeutics are widely employed for maintenance of cell viability, proliferation, and promotion of tissue regeneration [18, 19]. Controlled and localized delivery of these factors has been addressed to improve on site access to the cells in 3D microenvironment [18–22]. Insulin administration has demonstrated its ability to prolong the survival of chondrocytes and prevent the formation of necrotic cores inside the 3D collagen hydrogel construct [4, 17]. Furthermore insulin has its structural similarity to IGF-1 and may bind to the IGF-1 receptor to elicit similar effect on cartilage. This suggests that insulin can be an appropriate and inexpensive alternative growth factor to improve cartilage regeneration in 3D porous scaffolds [22]. Therefore development of suitable porous scaffold having good mechanical strength and controlled release of bioactive insulin for a prolonged duration is desirable for cartilage tissue engineering. Controlled and prolonged delivery of the insulin using PLGA microbeads prepared by water-in-oil-in-water (w-o-w) double emulsification technique has been demonstrated to be useful in cartilage tissue engineering [22]. Entrapment of these biodegradable microbeads carrying insulin within porous scaffolds of high mechanical strength may generate appropriate scaffolds for cartilage tissue regeneration.

In this research, we have made an attempt to prepare porous scaffolds with a controlled pore structure and controlled release of insulin as a bioactive 3D culture system for cartilage tissue engineering. The porous scaffold was prepared by spatial localization of insulin loaded PLGA microbeads in a 3D collagen porous scaffold by using a freeze-drying technique. Preprepared ice particulates having a diameter range of 150–250 *μ*m were used to control the pore structure of the scaffold. *In vitro* insulin release and degradation were studied over 4-week period at 37°C under shaking condition. Bovine articular chondrocytes were cultured in the hybrid scaffold to investigate the effect of released insulin on cell viability and proliferation.

## 2. Materials and Methods

### 2.1. Materials

PLGA (copolymer composition ratio of 50 : 50, weight average molecular weight of 20 kDa, and inherent viscosity of 0.187 to 0.229 dL/g), methylene chloride (CH_2_Cl_2_), polyvinyl alcohol (86–90 mol% hydrolysis), recombinant human insulin, hydrochloric acid (HCl), sodium hydroxide (NaOH), absolute ethanol (99.5%), N-hydroxysuccinimide esters (NHS), 25% glutaraldehyde solution, and sodium dihydrogen phosphate (NaH_2_PO_4_) were obtained from Wako Pure Chemicals Ltd., Japan. L-cysteine hydrochloride monohydrate (minimum 98%), ethylene diamine tetra acetic acid (EDTA), papain, DNA quantification kit, Dulbecco's Modified Eagle's Medium (DMEM), growth supplements, and antibiotics were obtained from Sigma-Aldrich, USA. Phosphate buffer saline (10x, pH = 7.4) was obtained from Nacali Tesque Inc., Japan. Porcine collagen type-1 was obtained from Nitta Gelatin, Japan. 1-Ethyl-3-[3-dimethylaminopropyl] carbodiimide hydrochloride (EDC/EDAC) was obtained from Peptide Institute Inc., Japan. Cellstain Double Staining Kit was obtained from Dojindo Laboratories, Japan. Micro BCA protein assay Kit was obtained from Pierce Biotechnology, USA. All the materials in this study were used as received without further purification. Molecular biology grade milli-Q water from millipore water system (Millipore Corporation, USA) was used for preparation of all the solutions and reagents.

### 2.2. Methods

#### 2.2.1. Insulin Microencapsulation

Insulin was microencapsulated in PLGA microbeads using w-o-w double emulsion technique [22–24]. PLGA solution at concentration of 0.5 g mL^−1^ was prepared by dissolving PLGA in methylene chloride. 50 *μ*L of insulin solution (insulin in 0.01 M HCl) at a concentration of 20 mg mL^−1^ (w_1_) was dispersed in 1 mL of PLGA (o) by homogenization at 8000 rpm for 1 minute. The resulted emulsion was further reemulsified in 2 mL saturated PVA (w_2_) prepared by mixing 1 : 1 (v/v) of 3% aqueous PVA and methylene chloride. The reemulsification process was carried out under high speed homogenization at 2000 rpm for 10 minutes. The double emulsion was added dropwise to 200 mL of 0.5% (w/v) PVA and stirred magnetically at 300 rpm in a hood for overnight to allow adequate solvent evaporation. The hardened microbeads were recovered after centrifugation (3500 rpm for 5 minutes). The microbeads were washed with milli-Q water and freeze-dried for 48 hours in a freeze drier (Vir Tis AdVantage Benchtop Freeze Dryer, S P Industries Inc., Japan) below 5 kPa to obtain dried insulin loaded PLGA microbeads.

#### 2.2.2. Preparation of Collagen-Microbead Hybrid Porous Scaffold

The collagen-microbead hybrid porous scaffolds were prepared by a freeze-drying method using preprepared ice particulates of a diameter range of 150–250 *μ*m as porogen material [15]. Ice particulates were prepared by spraying pure water droplets into liquid N_2_ and stabilized at −15°C in a low temperature chamber (WT-201, ESPEC Corp., Osaka, Japan). The ice particulates of 150–250 *μ*m were selectively sieved using two testing sieves having mesh pores of 150 and 250 *μ*m (Tokyo screen co. ltd., Japan). Hybrid scaffold was prepared from 2.0% (w/v) collagen aqueous solution with a ratio of ice particulate to collagen as 50 : 50 (w/v). The collagen aqueous solution (2.2% (w/v)) was prepared by dissolving freeze-dried collagen in a mixture solution of acetic acid (0.1 M, pH 3.0) and 10% ethanol. Dried insulin loaded PLGA microbeads were dispersed in 10% ethanol to prepare a suspension. The microbead suspension was sonicated for 1 minute in an ultrasonic water bath (Branson Ultrasonic Corporation, USA) in order to ensure free dispersion of microbeads. 2.2% (w/v) collagen aqueous solution was mixed with the prepared microbead suspension at a ratio of 9 : 1 to prepare microbead dispersed collagen aqueous solution. The manipulation was carried out at 4°C and the mixture solution was stirred under magnetic stirring for 1-2 hour. The mixture solution was transferred to a low temperature chamber maintained at −5°C and stirred magnetically for 2 hours for temperature balance. Ice particulates were added to the collagen-microbead mixture solution and mixed thoroughly to prepare a homogeneous ternary mixture of ice particulates, collagen, and microbeads. The final mixture was molded in a 5 mm thick silicone frame template. The mold was freeze-dried for 72 hours in a freeze drier. The freeze-dried scaffolds were cross-linked in 50 mM EDC and 20 mM NHS in 90% (v/v) ethanol for 24 hours at room temperature (RT). The cross-linked scaffolds were washed with milli-Q water and subjected to second freeze drying to prepare the final hybrid scaffolds. Control collagen porous scaffold was also prepared by using the similar procedure without the use of microbeads.

#### 2.2.3. Scanning Electron Microscopy (SEM)

The morphology of insulin loaded microbeads and scaffold microstructure was examined using a scanning electron microscope (SEM, JSM-5610, JEOL Ltd., Tokyo, Japan). Freeze-dried microbeads were dispersed over a carbon adhesive mounted over a copper stub and were sputtered with a thin layer of platinum by a sputter-coater (ESC-101, Elionix, Tokyo, Japan) for 500 seconds. The freeze-dried collagen scaffolds were cut into cross-sections and mounted on a carbon adhesive over the SEM stub. The cross-sections were sputter-coated with platinum for 300 seconds. The microbeads and scaffold cross-sections were observed at an acceleration potential of 5 kV and 10 kV, respectively.

#### 2.2.4. Microbead Size Analysis

10 mg of freeze-dried microbeads was suspended in 1 mL of milli-Q water and sonicated for 30 seconds using an ultrasonic water bath. The samples were analyzed for their average size as well as size distribution profile using a laser diffraction particle size analyzer (SALD 7000, Shimadzu Corporation, Japan). The microbead size was measured for three batches of formulation and the mean average was calculated as mean ± standard deviation (*n* = 3).

#### 2.2.5. Insulin Loading Efficiency (LE)

The confirmation of insulin loading as well as insulin quantification in microbeads was carried out using Micro-BCA Protein Assay Kit. 10 mg of dried insulin incorporated microbeads was dissolved in 1 mL of methylene chloride at RT. Insulin was extracted into 0.01 M HCl under vigorous shaking for 2 minutes in a high speed vortex device (Vortex Genie, Fischer, Pittsburgh, PA) at a setting of 10. The suspension was allowed to settle for 5 minutes at RT and supernatant aqueous phase containing insulin was extracted. 150 *μ*L of the supernatant solution was used for the insulin quantification. Briefly 150 *μ*L of albumin (BSA) standard in duplicate, and individual samples in triplicate, was added to individual wells of a 96-well plate. 150 *μ*L of assay working agent was added to each of the wells containing standards as well as samples. The plate was covered with a sealing tape and the mixing of the solutions in the wells was ensured by shaking the plate in a microplate shaker for 30 seconds. The microplate was incubated at 37°C for 2 hours. Following the incubation period, the plate was cooled at RT and the absorbance was measured at 562 nm by a microplate reader (Bio-Rad Laboratories, USA). The blank absorbance was subtracted and the insulin concentration (*μ*g/mL) of the unknown samples was measured by comparing with a standard curve (*R* ^2^ = 0.998) obtained from BSA standards. Total quantity of the incorporated insulin was calculated. The LE was calculated using following equation [23]:

(1) LE(%) =[Weight  of  the  incorporated  insulinweight  of  total  insulin  used  for  incorporation]  ×100.

#### 2.2.6. Mechanical Strength: Compression Test

Compression test was performed for evaluation of mechanical strength of the prepared scaffolds. The control collagen scaffold and collagen-microbead hybrid scaffold were cut into discs of a dimension of *Ø* 6 mm × H 4 mm and compressed with a texture analyzer (TA.XTPlus, Texture Technologies Corp., USA) at a rate of 0.1 mm/s. Young's modulus was calculated from the initial linear region of stress-strain relationship. The data was expressed as mean ± SD (*n* = 4).

#### 2.2.7. *In Vitro* Insulin Release and Microbead Degradation

*In vitro* insulin release from microbeads and hybrid scaffold was studied in PBS (pH = 7.4) at 37°C. 30 mg of microbeads was added in 2 mL tubes. Control collagen and collagen-microbead hybrid scaffolds were cut into discs of dimension of *Ø* 10 mm × H 5 mm and placed in 50 mL tubes. 1.2 mL of sterile PBS was added to the tubes containing microbead and 1 mL PBS was added to the tubes containing scaffolds. The tubes were tightly capped and incubated in a shaking water bath incubator (Taitec Corporation, Japan) at 37°C with a shaking speed of 50 rpm. The scaffolds were degassed before incubation to ensure the entry of PBS into the scaffold pores. After predetermined time points of 1, 2, 4, 8, 12, 16, 20, 24, and 28 days, the required volume of release medium (1 mL from microbeads and 0.5 mL from scaffolds) was collected and replaced with equivalent volume of fresh PBS. The insulin amount in released medium was quantified by Micro BCA protein assay and the cumulative release (%) was plotted against time to obtain the release curve. The experiments were performed in triplicate and data points in the curve were presented as mean ± standard deviation. The microbeads and scaffolds after 1-, 2-, and 4-week release period were collected and washed with milli-Q. The microbeads and scaffolds were freeze-dried. The freeze-dried samples were weighed for determination of dry weight of remaining microbeads. The remaining microbead weight (%) was plotted against the time to obtain the weight loss profile. The freeze-dried microbeads and cross-sections of scaffolds were observed under SEM.

#### 2.2.8. *In Vitro* Chondrocyte Culture

The control collagen scaffolds and collagen-microbead hybrid scaffolds were used for culture of bovine articular chondrocytes (BAC). The scaffolds were cut into discs of a dimension of *Ø* 6 mm × H 3 mm and sterilized with 70% ethanol. The sterile scaffolds were transferred to a clean bench, washed with PBS and incubated with cell culture medium for 3 hours in a CO_2_ incubator (Sanyo Corporation, Japan) equilibrated with 5% CO_2_ at 37°C. Bovine articular chondrocytes isolated from articular cartilage from the knees of a 9-week-old female calf were cultured in 75 cm^2^ tissue culture flasks in Dulbecco's Modified Eagle's Medium (DMEM) containing 10% fetal bovine serum, 4500 mg/L glucose, 4 mM glutamine, 100 U/mL penicillin, 100 *μ*g/mL streptomycin, 0.1 mM nonessential amino acids, 0.4 mM proline, 1 mM sodium pyruvate, and 50 *μ*g/mL ascorbic acid. The cells were harvested by treatment with trypsin/EDTA solution after 80% confluence. Cells were seeded in the scaffolds by dispensing 80 *μ*L of cell suspension with 7.5 × 10^5^ cells/scaffold. The cell-scaffold constructs were incubated for 3 hours in a CO_2_ incubator to allow cell adhesion. Following cell adhesion, the cell-scaffold constructs were transferred to new tissue culture plates and 10 mL cell culture medium was added. The cells were cultured for 1 week. Cell culture medium supplemented with 100 nM insulin was added to the wells containing control collagen scaffold as a positive control. Culture medium was changed twice a week. Cell seeding efficiency in the scaffolds was evaluated by counting the nonadhered cells using a hemocytometer as following equation:

(2) Cell  seeding  efficiency  (%) =[(number  of  seeded  cells−  number  of  nonadhered  cells  to  the  scaffold)×(number  of  seeded  cells)−1]×100.

The cell-scaffold constructs after 3 hours and 1 week of cell culture were fixed with 0.01% glutaraldehyde at RT. The fixed constructs were washed with milli-Q, freeze-dried and observed for cell adhesion and distribution using SEM.

Cell viability was evaluated by performing live-dead staining assay using Cellstain Double Staining Kit. After 1 week of cell culture, cell-scaffold constructs were washed with PBS and incubated in 2 *μ*M calcein-AM and 4 *μ*M propidium iodide solution in cell culture medium for 10 minutes. The cell seeding surface layer was cut and removed. The inner cross-section of specimen at approximate depth of 1 mm below the seeding surface was observed for live and dead cells using a fluorescence microscope (Olympus Corp., Japan).

The cell proliferation in the scaffolds was evaluated by quantifying the DNA amount in cell-scaffold constructs after 1-, 3-, and 7-day culture period. At each time point, the cell-scaffold constructs were collected, washed, and freeze-dried. The freeze-dried cell-scaffold constructs were digested with papain solution. Papain was dissolved at 400 *μ*g mL^−1^ in 0.1 M phosphate buffer (pH = 6.0) prepared with sodium dihydrogen phosphate, L-cysteine hydrochloride monohydrate, and ethylene diamine tetra acetic acid (EDTA). 500 *μ*L of papain solution was added to each aliquot containing freeze-dried cell-scaffold construct. The aliquots were incubated in a shaking incubator at 60°C with shaking speed of 150 rpm (24 hours) for complete digestion. The digested samples were used to measure the DNA content by using a standard curve (*R* ^2^ = 0.999) prepared using calf thymus DNA standard and fluorescence dye (Hoechst 33258). The fluorescence emission was measured using FP-6500 spectrofluorometer (JASCO, Japan) at an excitation wavelength of 360 nm and emission wavelength of 460 nm. Four samples were used to calculate the average and SD (*n* = 4).

#### 2.2.9. Statistical Analysis

All data were expressed as the mean ± standard deviation (SD). One-way analysis of variance was performed to reveal significant differences, followed by Tukey's post hoc test for pairwise comparison. Statistical analysis was executed using Kyplot 2.0 beta 15. The difference was considered significant when the *P* value was less than 0.05.

## 3. Results and Discussion

### 3.1. Morphology, Size Distribution, and LE of Insulin Loaded PLGA Microbeads

**Figure 1:**
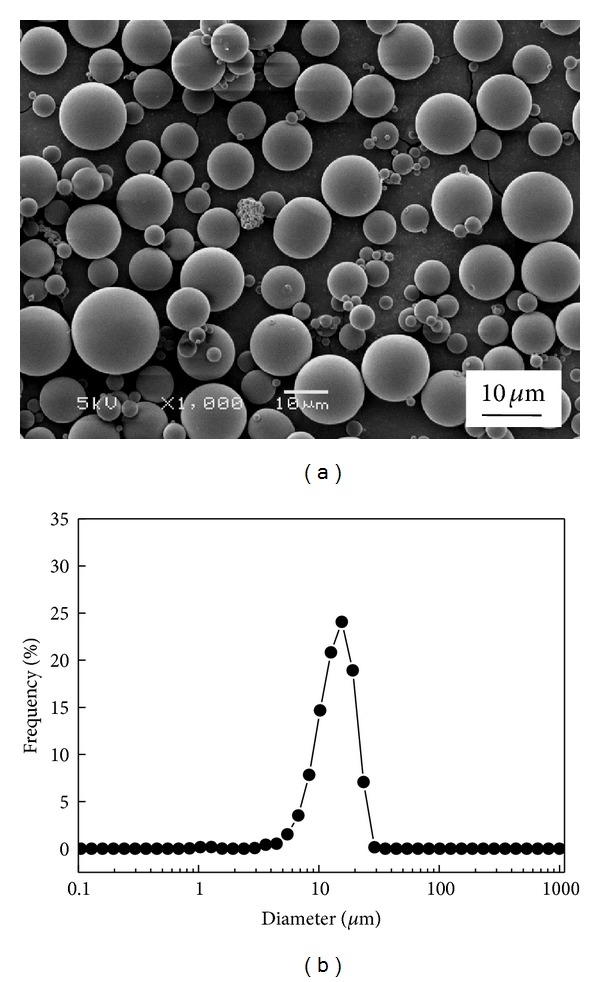
SEM photomicrographs of insulin loaded PLGA microbeads (a) size distribution of PLGA microbeads (b).

Human recombinant insulin was microencapsulated in PLGA microbeads using a w-o-w double emulsion technique.  Figure 1 shows the morphology and size distribution of the prepared microbeads. The microbeads were of spherical morphology with smooth surface without visible surface pores (Figure 1(a)). The microbeads showed narrow particle size distribution (Figure 1(b)). The mean diameter of the microbeads was 11.2 ± 0.9 *μ*m as determined using a laser particle size analyzer. The insulin LE of the microbeads was 67.3 ± 3.9%.

### 3.2. Scaffold Microstructure and Mechanical Strength

**Figure 2:**
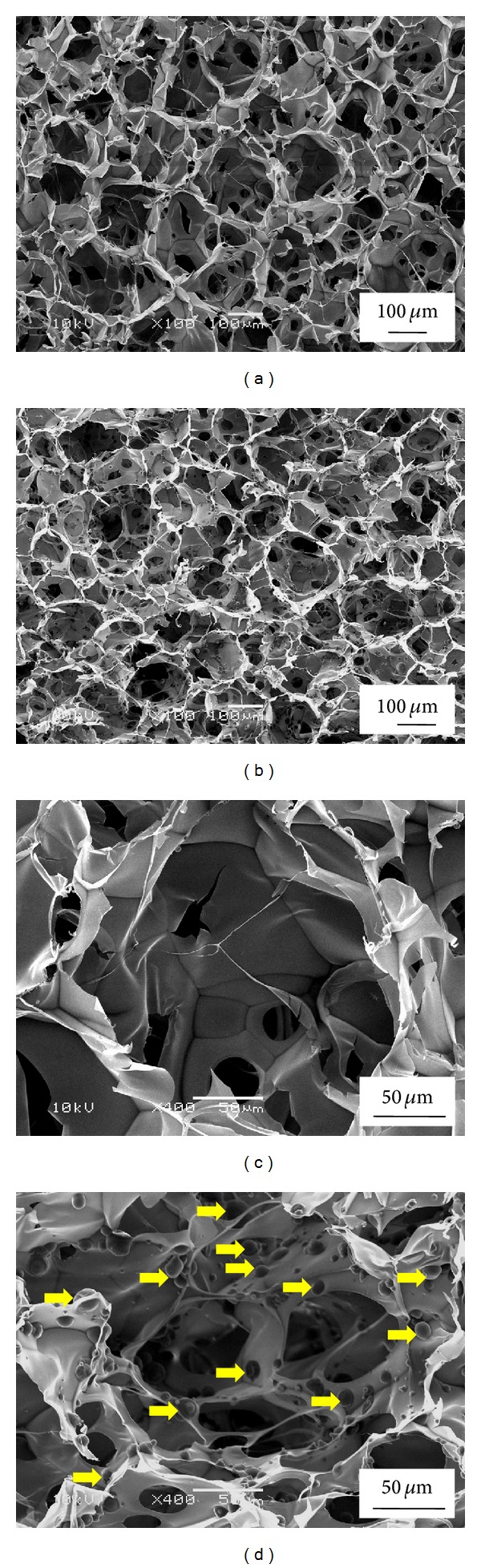
SEM photomicrographs of control collagen scaffolds (a), (c) and collagen-microbead hybrid porous scaffolds (b), (d) at low (a), (b) and high (c), (d) magnifications. Yellow arrows represent the integrated insulin loaded PLGA microbeads in porous collagen matrix.

Porous scaffolds with controlled pore structures were prepared by using preprepared ice particulates as a porogen material.  Figure 2 represents SEM microstructure of the control collagen scaffolds and collagen-microbead hybrid porous scaffolds. Figures 2(a) and 2(c) show the microstructure of control collagen scaffolds and Figures 2(b) and 2(d) show the microstructure of collagen-microbead hybrid scaffolds. All the scaffolds prepared with ice particulates had controlled pore structure and the large pores were replica of the ice particulates used during fabrication process. The large pores were connected to each other with interconnected small holes. The interconnected pores could facilitate cell migration, nutrient diffusion, and metabolic waste removal. The hybrid scaffolds exhibited a homogeneous spatial distribution of microbeads throughout the pore walls of the scaffold. The homogeneous and even distribution of microbeads could be useful for maintaining a uniform spatial release of insulin throughout the 3D microenvironment of hybrid scaffolds to meet the onsite insulin demand for the cultured chondrocytes.

**Figure 3:**
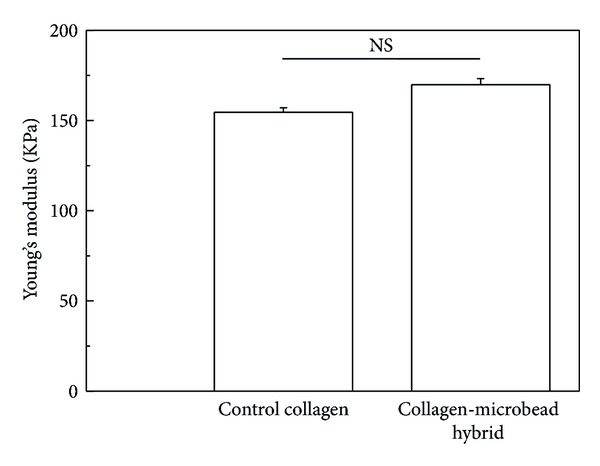
Compressive Young's modulus of control collagen and collagen-microbead hybrid scaffold. Data represent mean ± SD (*n* = 4), NS: no significant difference.

The mechanical strength of the scaffolds was determined using a compression test.  Figure 3 represents Young's modulus of the different scaffolds. The results indicated the control and collagen-microbead hybrid scaffolds had high mechanical strength, which was much higher than previously reported collagen scaffolds prepared without ice particulates [15]. Furthermore mechanical strength of hybrid scaffold was not compromised after the introduction of PLGA microbeads.

### 3.3. *In Vitro* Insulin Release and Microbead Degradation

**Figure 4:**
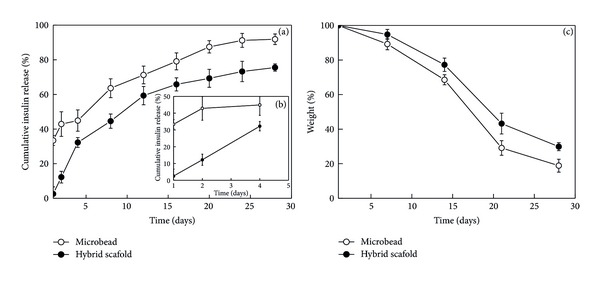
Cumulative insulin releases profile (a), (b) and weight loss profile (c) from free microbeads and hybrid scaffold for 4 weeks.

*In vitro* insulin release from microbeads and hybrid scaffold was studied for 4 weeks and the release profiles are shown in Figure 4. Figure 4(a) represents the cumulative release profile for entire 4 weeks and Figure 4(b) represents the release profile for a short time scale of 4 days. The release profile from microbeads showed a usual trend of initial burst release of 33% in the first day. The initial burst release was followed by a rise in cumulative insulin up to 3 weeks and a very slow release phase during the 4th week. However the hybridization of the microbeads in porous collagen scaffold avoided the initial burst release and a zero order release kinetics was achieved up to a period of 4 weeks. The suppression of insulin release from hybrid scaffold could be due to delayed induction of initial protein release from the microbeads inside the porous scaffolds [25, 26]. The result indicated entrapment of biodegradable microbeads containing insulin inside porous collagen matrix should be an effective strategy for long-term delivery of the insulin without initial burst release.

**Figure 5:**
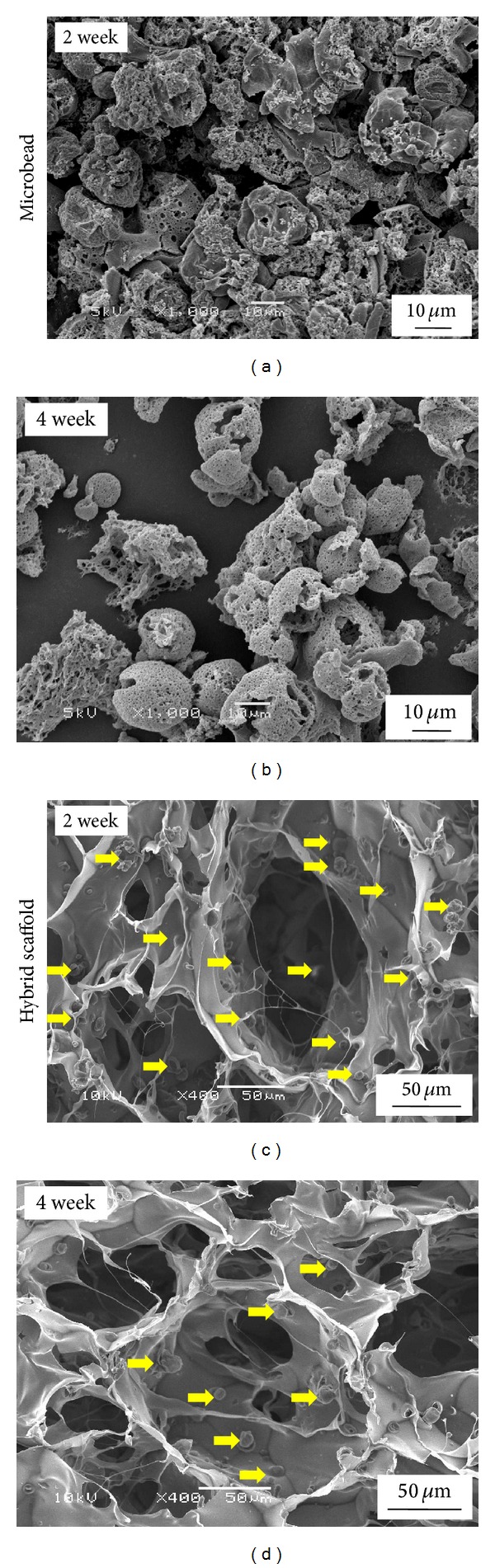
SEM photomicrographs of free microbeads and collagen-microbead hybrid scaffold after incubation for 2 and 4 weeks. Yellow arrows represent the degraded insulin loaded PLGA microbeads in porous collagen matrix.

Degradation of microbeads was studied using weight loss profile (Figure 4(c)). The weight loss profile demonstrated a quicker weight loss of microbeads in their free state compared to scaffold integrated state. This indicated degradation of microbeads was controlled after introduction to porous collagen matrix and showed their presence till the end of 4 weeks (Figure 5). Therefore a more controlled degradation of microbeads in collagen-microbead hybrid scaffolds could lead to a better control over the release of insulin and should sustain the release for a prolonged period.

### 3.4. Cell Adhesion, Viability, and Proliferation in Hybrid Scaffold

**Figure 6:**
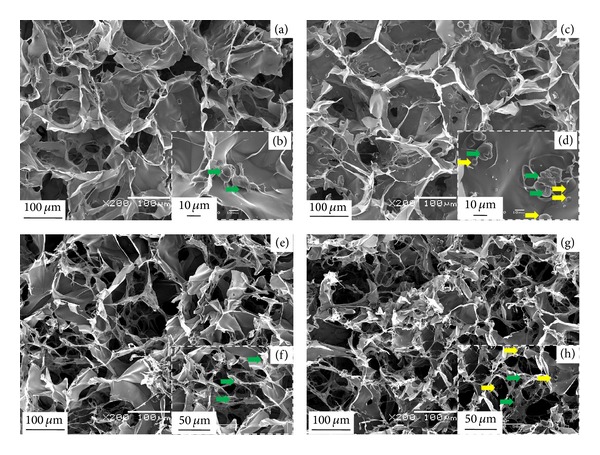
SEM photomicrographs of the cross-sections of control collagen (a), (b), (e), and (f) and collagen-microbead hybrid (c), (d), (g), and (h) scaffolds after 3 hours ((a)–(d)) and 1 week ((e)–(h)) of chondrocyte culture. Yellow arrows represent the integrated insulin loaded PLGA microbeads in porous collagen matrix and green arrows represent adhered chondrocytes.

Control collagen and collagen-microbead hybrid scaffolds were cultured with bovine articular chondrocytes. All the scaffolds were seeded with the cell suspension containing the same number of chondrocytes. The seeding efficiencies of control collagen and collagen-microbead hybrid scaffolds were 87.1 ± 1.1% and 87.0 ± 1.4%. Both control and hybrid scaffolds showed high cell seeding efficiencies. No significant difference in the seeding efficiency was observed among the scaffold groups indicating that microbead incorporation did not cause any difference in seeding efficiency. Figures 6(a)–6(d) present the SEM photomicrographs of cell adhesion and distribution at the inner cross sections of the scaffolds after 3 hours of culture. The cells adhered to the surface of the pore walls. Homogeneous cell distribution was observed at the entire inner cross-sections of the scaffolds, which indicated that the interconnected pore structure facilitated the cells to reach the inner pores in the scaffolds. Cells from the seeding surface could migrate into the inner bulk pores via interconnected pore structures and distributed in the entire scaffold. Figures 6(e)–6(h) show the SEM photomicrographs of inner cross-sections of the porous scaffolds after 1 week of cell culture. More cells were observed indicating cell proliferation in the scaffolds.

**Figure 7:**
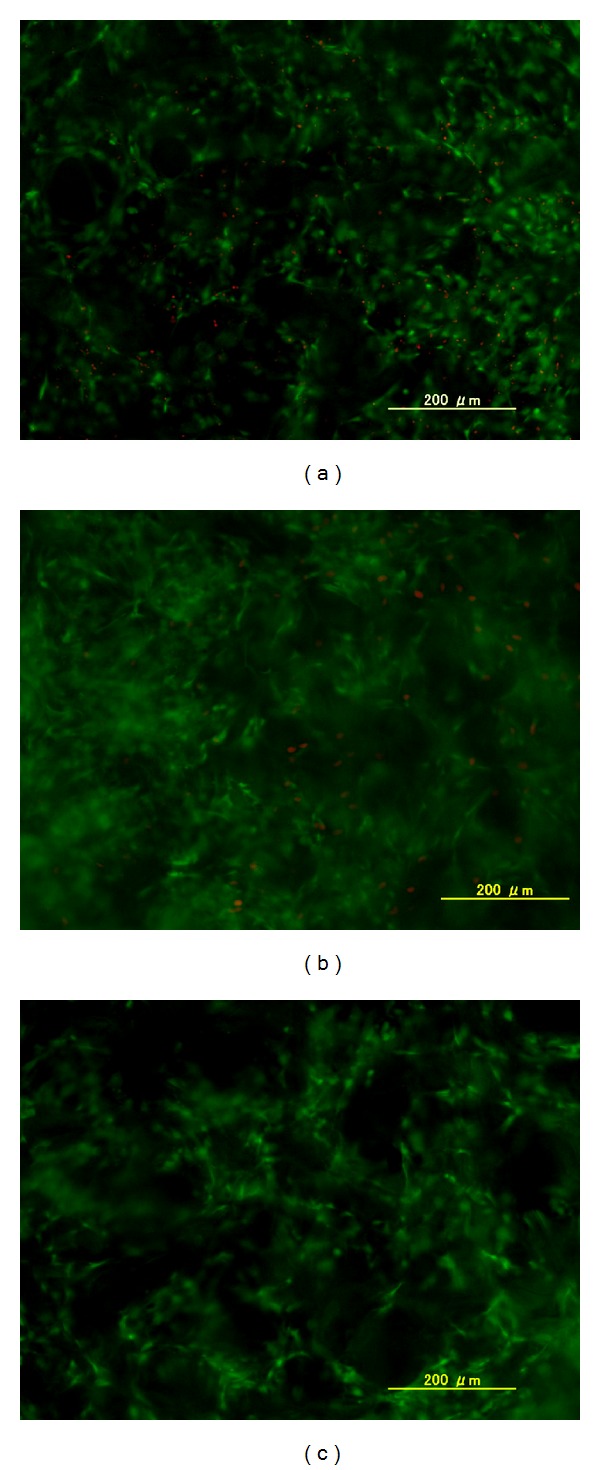
Live and dead staining of internal cross-sections of control collagen scaffold without insulin (a), control collagen scaffold with 100 nM insulin supplemented in medium (b), and collagen-microbead hybrid scaffold (c) after 1 week of chondrocyte culture. Green fluorescence indicates live cells and red fluorescence dots indicate dead cells.

Figure 7 represents cell viability at the inner cross-sections of different scaffolds after 1 week of cell culture. Fluorescence from the migrated cells was observed at an approximate depth of 1 mm below the seeding surface. Green fluorescence represents the live cells and red fluorescence dots indicate the dead cells. More dead cells were detected in control collagen scaffold without insulin supplement compared to collagen scaffolds supplemented with 100 nM external insulin and collagen-microbead hybrid scaffolds. This indicated insulin had some effect on maintenance of chondrocyte viability. Furthermore very few or no dead cells were detected from the collagen-microbead hybrid scaffolds. This might be due to the sustainable local concentration of released insulin from the hybrid scaffolds.

**Figure 8:**
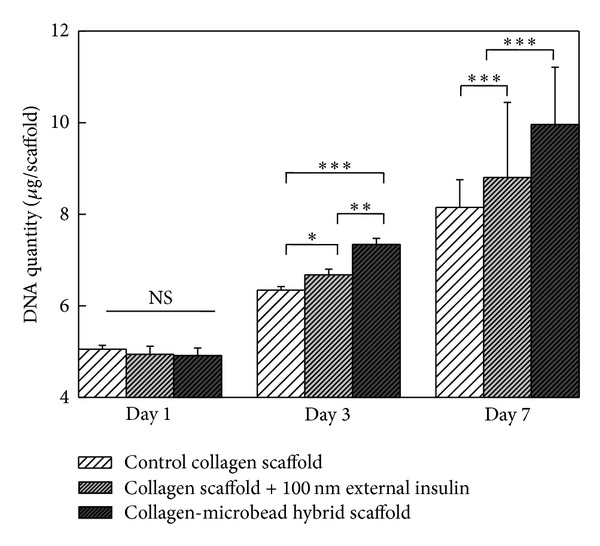
DNA amount in the cell/scaffold constructs of control collagen scaffold, control collagen scaffold supplemented with 100 nM insulin, and collagen-microbead hybrid scaffold after 1, 3, and 7 days of chondrocyte culture. Data represent mean ± SD (*n* = 4), *significant (*P* < 0.05), **significant (*P* < 0.01), ***significant (*P* < 0.001), NS: no significant difference.

Figure 8 shows the cell proliferation of different scaffolds. All the scaffolds showed an increased DNA amount during 7-day culture period. A significant difference in the cell number among the scaffold groups was noticed after 3 and 7 days of culture. Cell proliferation in the scaffolds showed an increase order of collagen control scaffold without insulin < control collagen scaffold with 100 nM external insulin supplement < hybrid scaffold. This result suggested that insulin promoted chondrocyte proliferation. The insulin released from the spatially located microbeads in the hybrid scaffolds might have met the local insulin demand for the chondrocytes for their survival and usual proliferation. However due to extensive cell proliferation with time, the diffusion of bioactive insulin from cell culture medium might not be sufficient to nourish the interior cells of the scaffold supplemented with external insulin. This suggested the unavailability or limited availability of insulin to the cells inside the control scaffolds might be the reason for low cell viability and proliferation.

The study depicted a controlled release approach to promote cell proliferation in 3D porous collagen for cartilage tissue engineering. Owing to the importance of collagen porous scaffolds of controlled pore structure and improved mechanical strength in cartilage tissue regeneration, controlled release function via biodegradable microbeads was additionally introduced in order to improve the regeneration potential of the prepared scaffold. The present approach suggested the possibility to use other bioactive growth factors as nutrients for cell survival in large and thick 3D porous scaffolds for tissue engineering and regenerative medicine.

## 4. Conclusion

A controlled release porous collagen-microbead hybrid scaffold having a controlled pore structure was prepared by introduction of insulin loaded PLGA microbeads into porous collagen sponge formed with preprepared ice particulates. The collagen-microbead hybrid scaffold demonstrated a high mechanical strength and a stable release of insulin for 4 weeks. The released insulin demonstrated its effect on cultured chondrocytes for their survival and proliferation. The bioactive hybrid scaffold should be useful for maintenance of prolonged survival and proliferation of cultured chondrocytes for application to cartilage tissue engineering.
